# Sirtuin-1 expression and activity is diminished in aged liver grafts

**DOI:** 10.1038/s41598-020-68314-6

**Published:** 2020-07-17

**Authors:** Uwe Scheuermann, Elisabeth R. Seyferth, Nader Abraham, Samuel J. Kesseli, Samantha E. Halpern, Minghua Zhu, Mingqing Song, Matthew G. Hartwig, William Parker, Jean Kwun, Anne D. Cherry, Jaewoo Lee, Andrew S. Barbas

**Affiliations:** 1 Department of Visceral, Transplantation, Vascular, and Thoracic Surgery, University Hospital of Leipzig, Leipzig, Germany; 2 Department of Surgery, Duke University, DUMC 3512, Durham, NC 27710 USA; 3 Department of Anesthesiology, Duke University, Durham, USA

**Keywords:** Liver diseases, Preclinical research

## Abstract

The cellular mechanisms underlying impaired function of aged liver grafts have not been fully elucidated, but mitochondrial dysfunction appears to be contributory. Sirtuin1 has been identified as a key mediator of mitochondrial recovery following ischemia–reperfusion injury. The purpose of this study was to determine whether differences exist in sirtuin-1 expression/activity in old vs. young liver grafts and to determine correlations with mitochondrial function, graft metabolic function, and graft injury. Old and young rat liver grafts (N = 7 per group) were exposed to 12 h of static cold storage (SCS), followed by a 2 h period of graft reperfusion ex vivo. Sirtuin1 expression and activity, mitochondrial function, graft metabolic function, and graft injury were compared. Sirtuin1 expression is upregulated in young, but not old, liver grafts in response to cold storage and reperfusion. This is associated with diminished tissue ATP, antioxidant defense, and graft metabolic function in old liver grafts. There was no evidence of increased inflammation or histologic injury in old grafts. Sirtuin1 expression is diminished in old liver grafts and correlates with mitochondrial and metabolic function. The sirtuin pathway may represent a target for intervention to enhance the function of aged liver grafts.

## Introduction

Although liver transplant (LT) outcomes are excellent in the modern era, the field continues to be limited by a shortage of suitable donor organs. Recent efforts have focused on expanding the donor pool by utilizing grafts from extended criteria donors (ECDs), including donors of advanced age^1^. Although LT outcomes using older grafts have improved over time^2,3^, multiple studies have identified advanced donor age as an independent risk factor in impaired graft outcomes^4–6^. These inferior clinical outcomes have been attributed to a reduced capacity of older grafts to recover from prolonged cold ischemia and ischemia–reperfusion (I–R) injury^7–10^. From a cellular perspective, mitochondrial dysfunction has been recognized as a central mediator of liver I–R injury, characterized by ATP depletion, reactive oxygen species (ROS) generation, and cell death^11–13^.

Recent studies have demonstrated the importance of sirtuin-1, an NAD^+^-dependent deacetylase, in mitochondrial recovery pathways following I–R injury of the liver^14–16^. These effects are attributed to the role played by sirtuin-1 in the clearance of damaged mitochondria (mitophagy), production of new mitochondria (biogenesis), and antioxidant defense^14,17–20^. Importantly, these mitochondrial recovery pathways appear to be diminished in old hepatocytes subjected to I–R injury. A recent study by Chun et al. demonstrated that in contrast to young hepatocytes, aged hepatocytes exhibited a near complete loss of sirtuin-1 following I–R, which corresponded with significantly increased injury^16^. Taken together, these studies highlight the importance of sirtuin-1 activity in the mitochondrial response to I–R injury, and indicate that differences in sirtuin-1 activity between young and old hepatocytes may contribute to differences in the severity of I–R injury.

While the aforementioned studies have established the role of sirtuin-1 in mitochondrial recovery from I–R, the impact of sirtuin-1 in liver transplantation remains unknown. The purpose of the present study was to determine whether differences exist in sirtuin-1 expression/activity in old vs. young liver grafts and to determine correlations with mitochondrial function, graft metabolic function, and graft injury. We hypothesized that old liver grafts would demonstrate reduced sirtuin-1 activity, impaired mitochondrial and metabolic function, and increased injury in comparison to young grafts. To test this hypothesis, old and young liver grafts were subjected to cold ischemic storage followed by ex vivo reperfusion. Measures of sirtuin-1 expression/activity, mitochondrial function, graft function, and graft injury were compared. We demonstrate that following IR, young liver grafts upregulate sirtuin-1 expression while old grafts do not. Furthermore, we demonstrate that sirtuin1 expression correlates with graft mitochondrial and metabolic function, without apparent differences in histologic injury.

## Methods

### Animals

Male Lewis rats (Charles River Laboratories, Wilmington, MA), and male August Copenhagen Irish (ACI) rats (Envigo, Indianapolis, IN) were used for the experiments. All animals were housed in specific pathogen-free conditions in the animal care facilities at the Duke University Medical Center in accordance with institutional guidelines. All animal care and procedures involving laboratory animals were approved by the Institutional Animal Care and Use Committee at Duke University.

### Study design

**Figure 1:**
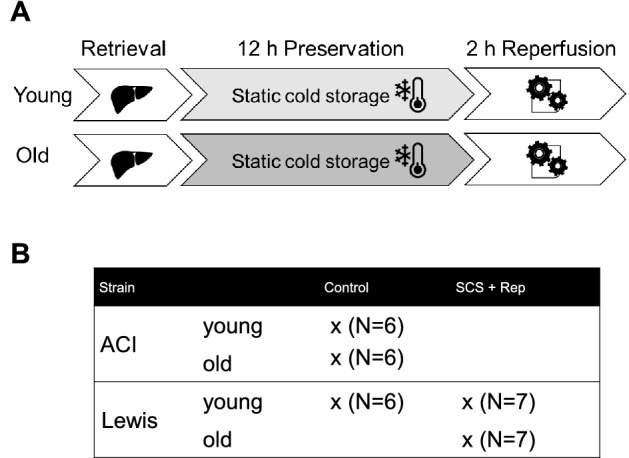
Experimental design and groups. **a** Livers from old (26 months) and young (2.5 months) Lewis rats were preserved by static cold storage (SCS) for 12 h, then reperfused ex vivo at 37 °C for 2 h using Krebs–Henseleit buffer to simulate transplantation. **b** Untreated livers from old and young ACI rats were used to assess for baseline age-related changes in gene and protein expression.

The study consisted of two experimental groups in which liver grafts were exposed to 12 h of cold storage followed by 2 h of ex vivo reperfusion: Old Lewis rats (26 months, N = 7) vs. young Lewis rats (2.5 months, N = 7). Three control groups consisting of untreated livers were also tested: old ACI rats (28 months, N = 6), young ACI rats (2.5 months, N = 6), and young Lewis rats (2.5 months, N = 6) (Fig. 1). Age and weights of the animals are shown in Table 1.

**Table 1:** Animal characteristics.

	ACI			Lewis		
	Young (N = 6)	Old (N = 6)	*p*	Young (N = 13)	Old (N = 7)	*p*
Age (months)	2.5 ± 0.1	27.9 ± 0.0	< 0.001	2.5 ± 0.0	26.0 ± 0.2	< 0.001
Body weight (g)	239 ± 4.2	356 ± 21.1	< 0.001	294 ± 4.2	692 ± 11.4	< 0.001
Liver weight (g)	7.9 ± 0.2	9.5 ± 0.5	0.007	10.1 ± 0.2	14.2 ± 0.3	< 0.001
Liver/ body weight (%)	3.3 ± 0.1	2.7 ± 0.1	< 0.001	3.4 ± 0.0	2.0 ± 0.1	< 0.001

Age, body, and liver weights of ACI and Lewis rats used in experiments. Data shown as mean ± SEM.

## Experimental procedures

### Liver procurement

As we previously described^21^, all surgeries were performed under cone mask anesthesia with continuous 5% isoflurane (Isothesia, Henry Schein Animal Health, Melville, NY) for induction, and 2–3% isoflurane during the procedure, with 2 L/minute oxygen flow. The abdominal cavity was opened by a midline and transverse incision. A stent fashioned from a 24-gauge angiocatheter (BD Insyte autoguard Becton Dickinson, Franklin Lakes, NJ) was inserted into the common bile duct and secured. The proper hepatic artery and gastrosplenic and duodenopancreatic branches of the portal vein were isolated and divided. Heparin (1 IU/g bodyweight, Fresenius Kai, Lake Zurich, IL) was injected through the infrahepatic vena cava with a 30-gauge needle. Then, 5 min later, the portal vein was cannulated with a perfusion cannula (Harvard Apparatus, Holliston, MA), and the liver gently flushed with 40 mL of cold University of Wisconsin (UW) solution (Bridge of Life, Columbia, SC). The liver was then explanted and weighed.

### Static cold storage

As we previously described^21^, for static cold storage (SCS), grafts were immersed in 50 mL UW solution (Bridge of Life) at 4 °C for 12 h. At different time points, livers were flushed with 10 mL UW solution. 1.5 mL flush solution was centrifuged at 2,500 rpm at 4 °C for 15 min to remove cellular debris. The supernatant was transferred to 1-mL Corning cryogenic vials immediately after centrifugation, frozen with liquid nitrogen and stored at − 80 °C for subsequent analysis.

### Ex vivo graft reperfusion

As we previously described^21^, after cold storage preservation, grafts were transferred to the perfusion circuit and reperfused ex-vivo for 2 h. The reperfusion was performed in a recirculating system consisting of a perfusate reservoir, an autoclavable organ chamber (Type 834/10), an oxygenator, a peristaltic pump and a bubble trap (Hugo Sachs Elektronik, March-Hugstetten, Germany). The chamber was enclosed in a conditioning system, which allows precise regulation and control of the temperature (Optima T100, Grant instruments, Beaver Falls, PA). Perfusion media consisted of 100 mL oxygenated (95% O_2_ and 5% CO_2_; Airgas, Durham, NC) Krebs–Henseleit buffer (Sigma Aldrich, St. Louis, MO) supplemented with 5% bovine serum albumin (Hyclone, GE Healthcare Life Sciences, South Logan, UT), and 100,000 U/L penicillin, 100 mg/L streptomycin (Gibco-Life Technologies, Grand Island, NY). Perfusate flow rate was 3 mL/minute/g-liver, and temperature was maintained at 37 °C. Before reperfusion, the liver was flushed with 20 mL 0.9% normal saline at room temperature and allowed to equilibrate for 15 min to simulate the performance of vascular anastomoses (equilibration phase).

## Analytical methods

### Intrahepatic vascular resistance

As we previously described^21^, the intrahepatic vascular resistance was calculated as portal vein pressure (mmHg)/perfusate flow rate (mL/minute). Vascular resistance ratio was calculated as the vascular resistance at a specific time point divided by the initial vascular resistance. During reperfusion, portal vein pressure was measured continuously (Flow pressure transducer P75, HSE amplifier module TAM-D; Hugo Sachs Elektronik). LabChart Pro software (AD Instruments, Colorado Springs, CO) was used to display and record flow rates and portal vein pressure.

### Blood gas analysis

As we previously described^21^, perfusate samples were drawn from the portal venous inflow and from the suprahepatic caval outflow at different time points for blood gas analysis using a point-of-care device (iSTAT, CG4 + cartridges, Abbott Point of Care Inc., Abbott Park, IL). Measurements included lactate concentration and acid–base parameters (pH, HCO_3_-, base excess, pO2, and pCO2).

### Calculation of oxygen consumption rate

As we previously described^21^, the oxygen consumption rate during reperfusion was calculated using the following equation: Oxygen consumption rate (μL/minute/g-liver) = perfusate flow x S x (P_i_O_2_—P_o_O_2_)/g-liver. In this equation, P_i_O_2_ represents the partial pressure of oxygen of the inflow (portal vein), P_o_O_2_ represents the partial pressure of oxygen of the outflow (suprahepatic vena cava), and S represents the solubility constant of oxygen in water at 37 °C (0.031 μL/mL/mmHg).

### Bile processing and analysis

As we previously described^21^, bile was collected throughout reperfusion and volume was determined hourly. Bile was transferred to 1-mL Corning cryogenic vials (VWR, Atlanta, GA), frozen with liquid nitrogen and stored at – 80 °C for subsequent analysis. Biliary lactate dehydrogenase (LDH) was determined using ELISA (LifeSpan BioSciences, Seattle, WA, USA), and biliary glucose was analyzed with the iSTAT point-of-care device (Chem8 + cartridges; Abbott Point of Care).

### Perfusate analysis

As we previously described^21^, at different time points, 2 mL of perfusate was collected and centrifuged at 2,500 rpm at 4 °C for 15 min to remove cellular debris. The supernatant was transferred to 1-mL Corning cryogenic vials immediately after centrifugation, frozen with liquid nitrogen, and stored at – 80 °C for subsequent analysis.

### Liver damage parameters

Alanine aminotransferase (ALT) in perfusate was measured using a Piccolo Xpress Chemistry Analyzer (Abaxis, Union City, CA). LDH was determined using ELISA (LifeSpan BioSciences).

### DAMP levels

As we previously described^21^, levels of extracellular DNA (exDNA) were determined using Quant-iT PicoGreen double-stranded DNA kit (Molecular Probes, Eugene, OR). High-Mobility Group Box 1 (HMGB1) levels were determined by ELISA (IBL, Hamburg, Germany).

### Toll-like receptor activation

As we previously described^21^, TLR activation was measured using TLR reporter cell lines human embryonic kidney (HEK)–hTLR3, HEK-hTLR4, and HEK-hTLR9 cells (InvivoGen, San Diego, CA), stably expressing a nuclear factor kappa B (NF-κB)/activator protein 1-inducible secreted embryonic alkaline phosphatase (SEAP). All cells were grown in Dulbecco’s modified Eagle’s medium supplemented with 10% fetal bovine serum at 37 °C in a humidified atmosphere with 5% CO2. Perfusates were diluted to 15% (vol/vol) in growth media and incubated with TLR reporter cells in flat-bottom 96-well plates for 16 h. SEAP levels were then determined by colorimetric assay using QUANTIBlue assay (InvivoGen). Polyinosinic: polycytidylic acid (5 μg/mL, InvivoGen), lipopolysaccharide (20 ng/mL, Sigma-Aldrich), and CpG (1 mM, InvivoGen) were used as TLR3, TLR4, and TLR9 stimulator controls, respectively. Culture media from stimulated HEK-null cell lines (InvivoGen) were subtracted from experimental samples to account for endogenous levels of alkaline phosphatase.

### Cytokine levels

The concentrations of interleukin (IL) 1α, IL2, IL4, and tumor necrosis factor α (TNF-α) in perfusate samples were measured by flow cytometry using BD Cytometric Bead Array Rat Flex sets (BD Bioscience, San Jose, CA) on a BD LSRFortessa X-20 analyzer (BD Bioscience).

### Liver tissue processing

Liver parenchyma was partitioned in three ways for assays. A portion of tissue was flash-frozen in liquid nitrogen and stored at − 80 °C for genomic DNA isolation, analysis of ATP concentration, ADP/ATP ratio, and sirtuin1 activity. Another portion of tissue was immersed in RNAlater Solution (Thermo Fisher Scientific, Waltham, MA, USA) and frozen at − 80 °C for later RNA isolation. A final portion of tissue was fixed in 10% formalin for histologic assessment.

### RNA extraction and quantitative polymerase chain reaction

As we previously described^21^, total RNA from frozen liver samples was isolated using the QIAshredder and RNeasy Mini-Kit (Qiagen, Hilden, Germany). The resultant RNA was quantified using NanoDrop 2000 Spectrophotometer (Thermo Fisher Scientific). Subsequently, equal amounts of RNA were converted to cDNA with the iScript complementary DNA Synthesis Kit (Biorad, Hercules, CA, USA). Taq-Man gene expression assays (Applied Biosystems, Foster City, CA, USA) were performed using the following primers: 18S ribosomal RNA (Rn18s; Rn03928990_g1), glyceraldehyde-3-phosphate dehydrogenase (GAPDH; Rn01775763_g1), heme oxygenase 1 (HMOX-1; Rn00561387_m1), hypoxanthine phosphoribosyltransferase 1 (HPRT1; Rn01527840_m1), interleukin-6 (IL-6; Rn01410330_m1), parkin RBR E3 ubiquitin protein ligase (Park2; Rn00571787_m1), succinate dehydrogenase complex flavoprotein subunit A (SDHA; Rn00590475_m1), sirtuin-1 (SIRT1; Rn01428096_m1), superoxide dismutase 1 (SOD-1; Rn00566938_m1), superoxide dismutase 2 (SOD-2; Rn00690588_g1), and tumor necrosis factor alpha (TNF-α; Rn99999017_m1). All samples were analyzed in triplicate. NormFinder^22^, geNorm^23^, and BestKeeper^24^ software were used to measure stability of candidate reference genes GAPDH, SDHA, Rn18s, and HPRT1. The stability measurements were combined to establish a consensus rank of the genes. The combination of genes HPRT1 and Rn18s resulted in the lowest variability and mean of both genes were used as endogenous control for gene expression analysis.

### MtDNA/nDNA ratio

Genomic DNA was extracted from liver samples (25 mg) with GenElute Mammalian Genomic DNA Miniprep Kit (Sigma-Aldrich) according to manufacturer’s protocol. Analysis of mtDNA/nDNA ratio was performed with quantitative polymerase chain reaction using PrimePCR SYBR Green Assays (mitochondrial DNA: NADH dehydrogenase subunit 1 (ND1), Cytochrome B (CytB); nuclear DNA: GAPDH; Biorad, Hercules, CA) and SsoAdvanced Universal SYBR Green Supermix (Biorad).

### Western blot analysis

As we previously described^21^, rat livers either freshly isolated or following graft reperfusion were frozen in RNAlater (Thermo Fisher Scientific) at – 80 °C. Protein from liver pieces was then extracted using RIPA lysis buffer (150 mM sodium chloride, 1% Triton X-100, 0.5% sodium deoxycholate, 0.1% SDS, 50 mM Tris, pH 8.0). 10–20 μg of total protein from each sample was separated on SDS-PAGE and transferred to a nitrocellulose membrane. Antibodies used for western blotting were the following: anti-beta actin antibody (sc-47778, Santa Cruz Biotechnology, Dallas, TX), anti-PGC-1α (ab54481; Abcam), anti-SDHA (ab14715; Abcam), anti-GAPDH (ab181602; Abcam), and anti-NAD-dependent deacetylase Sirtuin-1 (SIRT1; ab189494; Abcam). Bound antibodies on membranes were removed using Restore Plus Western Blot Stripping Buffer (Thermo Fisher Scientific) when additional western blotting was needed.

### Analysis of ATP concentration and ADP/ATP ratio

Frozen liver tissue was weighed on ice, crushed in liquid nitrogen, and rapidly homogenized in ice–cold 0.1 M phosphate buffer solution (Sigma-Aldrich). Phosphate buffer solution was adjusted to pH 8.0 using 6 N Sodium hydroxide. This homogenate was immediately transferred to 1 volume 2.5% trichloroacetic acid (TCA, Sigma Aldrich) and centrifuged at 3,000 × g for 15 min at 4 °C.

An aliquot of the supernatant was diluted 1:1 in 100 mmol/L phosphate buffer (pH 8.0) for use in determination of protein concentration using Pierce BCA Protein Assay Kit (ThermoScientific, Rockford, IL). Another aliquot (1:1) was used for analysis of ATP concentration (ATP Bioluminescence Assay Kit CLS II, Sigma-Aldrich) content following manufacturer’s protocol under a luminometer (TECAN GENios Microplate reader) in a white 96-well microplate (Corning). ATP concentrations was calculated with a calibration curve constructed on the same plate and corrected for amount of protein (value expression as µmol/g protein). ADP/ATP Ratio was analyzed using bioluminescent detection (ADP/ATP Ratio Bioluminescent Assay Kit; ab65313; Abcam) according to the manufacturer’s protocol. Therefore, samples were diluted 1:3, and plated in white 96-well microplates before bioluminescent intensities were measured.

### Sirtuin-1 enzyme activity

10 mg liver tissue was mashed through a cell strainer with a 70 μm pore size (Corning) and syringe plunger. Liver homogenates were dissolved in lysis buffer (10 mM Tris HCl (pH 7.5), 10 mM NaCl, 15 mM MgCl_2_, 250 mM sucrose, 0.5% NP-40, and 0,1 mM EGTA) and kept on ice for 15 min. Then, cell lysates were spun through 4 mL 30% sucrose solution containing 10 mM Tris HCl (pH 7.5), 10 mM NaCl, and 3 mM MgCl_2_ at 1,300 g for 10 min at 4 °C. Pellets were gently re-suspended and washed with cold 10 mM Tris–HCl (pH 7.5) containing 10 mM NaCl, then re-suspended in 100 μL extraction buffer consisting of 50 mM Hepes KOH (pH 7.5), 420 mM NaCl, 0.5 mM EDTA, 0.1 mM EGTA Na_2_, and 10% glycerol and transferred into 1,5-mL microtubes. After sonication for 30 s, isolated nuclei were put on ice for 30 min and then spun at 20,000 g for 10 min. Supernatant containing crude nuclear extracts were transferred into new microtubes and stored at – 80 °C for subsequent analysis. Sirt1 deacetylase activity was quantified in nuclear extracts with SIRT1 Activity Fluorometric Assay Kit (ab156065; Abcam) following manufacturer’s protocol. 15 μL of enzyme samples were used per assay reagent. Fluorescence intensity (excitation 340 nm, emission 460 nm) was measured at 1-min intervals for 60 min in a microplate reader (SpectraMax i3). The results are reported as relative fluorescence/mg of protein. Protein concentrations were determined using Pierce BCA Protein Assay Kit (ThermoScientific).

### Histology

Liver tissue was fixed in 10% formalin and paraffin embedded. Sections were obtained from the medial left lateral lobe. Paraffin sections were stained with hematoxylin and eosin (H&E) for morphologic observation. Severity of histological damage was blindly scored by Suzuki criteria after H&E staining. Sinusoidal congestion, hepatocyte necrosis, and ballooning degeneration were graded from 0 to 4 points, and the final score is the sum of the grades for each item as previously described^25^. Immunohistochemical (IHC) staining for NRF-1 (Abcam) was used to demonstrate the expression of NRF-1 in the cells. Apoptotic cells were identified by terminal dUTP nick-end labeling of fragmented DNA assay (TUNEL) (Roche, Mannheim, Germany). Whole slide digital images were captured by the Aperio AT Turbo digital slide scanner system (Leica Biosystems, Vista, CA). Quantitative immunohistochemical analysis was performed using Aperio Imagescope digital pathology software (Leica Biosystems). NRF-1 expression and TUNEL stain level were analyzed using an optimized positive pixel algorithm to obtain a percent pixel positivity of in the measured areas.

### Transmission electron microscopy

Two livers from both experimental groups were perfused through the portal vein with 2% Glutaraldehyde/ 2% Paraformaldehyde in Phosphate Buffer, pH 7.4 (Electron Microscopy Sciences, Hatfield, PA). After fixation, samples of the left lateral liver lobe were stored in the same fixation solution at 4 °C before analysis.

### Statistical analysis

Graphpad Prism software, version 7.04 (GraphPad Software Inc., La Jolla, CA) was used for statistical analyses and graphs. All values are presented as mean ± standard error of the mean (SEM). Differences between continuous values were analyzed using unpaired Student *t* test. The Mann–Whitney U test was used for evaluation of the Suzuki score. *p *Values < 0.05 were considered significant.

## Results

### Sirtuin1 expression is upregulated in young, but not old, liver grafts in response to cold storage and reperfusion

**Figure 2:**
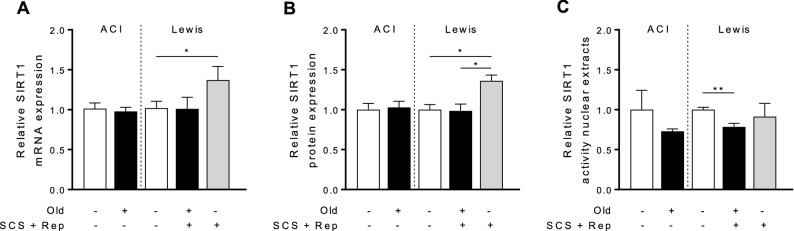
SIRT1 expression and activity. Tissue samples from old and young liver grafts following cold storage and reperfusion were assessed to determine gene and protein expression of Sirt1 and enzyme activity of Sirt1 by fluorometric activity assay. **a** Sirt1 mRNA expression is significantly higher in young liver grafts compared to untreated controls, while this difference is not observed in old grafts. **b** Sirt1 protein expression is significantly higher in young liver grafts vs. old liver grafts and untreated controls. **c** Sirt1 enzyme activity level is diminished in old liver grafts bit maintained in young liver grafts. Data shown as mean ± SEM, N = 3–5 per group, *p < 0.05, **p < 0.01.

Sirtuin1 mRNA expression was significantly upregulated in young liver grafts in response to ischemia–reperfusion, while this was not observed for old grafts (Fig. 2a). Similarly, sirtuin1 protein levels were significantly increased in young liver grafts in comparison to both old liver grafts and untreated control liver tissue (Fig. 2b). Sirtuin1 enzyme activity was diminished in old liver grafts compared to control young liver tissue, which was not observed for young grafts (Fig. 2c).

### Tissue ATP is lower in old liver grafts despite similar mitochondrial mass

**Figure 3:**
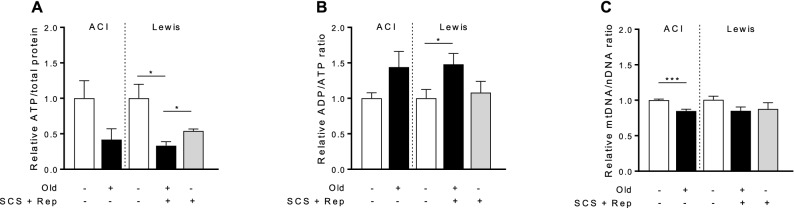
Tissue energy status and mitochondrial mass in old versus young livers. Tissue samples from old and young liver grafts following reperfusion were assessed to determine ATP and ADP levels. Mitochondrial mass was assessed by measuring mitochondrial DNA using quantitative PCR. Data are normalized to untreated control tissue from young rats. **a** Tissue ATP levels are significantly lower in old liver grafts following reperfusion, indicative of impaired mitochondrial function. **b** Similarly, ADP: ATP ratio is significantly higher in old liver grafts following reperfusion compared to control liver tissue, reflecting depletion energy substrate. **c** MtDNA levels (ND1: GAPDH) are not significantly different between old and young liver grafts following reperfusion, indicating similar mitochondrial mass. Control liver tissue from old ACI rats demonstrated significantly lower mtDNA levels at baseline compared to young ACI rats (left panel). Data shown as mean ± SEM, N = 4–6 per group, *p < 0.05, ***p < 0.001.

Mitochondrial function was impaired in old liver grafts after reperfusion, as evidenced by lower tissue ATP levels and an elevated ADP: ATP ratio (Fig. 3a, b). Mitochondrial mass was lower in untreated old vs. young livers at baseline (Fig. 3c). However, in the experimental groups, there were no significant differences in mitochondrial mass observed for young and old liver grafts following reperfusion.

### Antioxidant gene expression is impaired in old liver grafts

**Figure 4:**
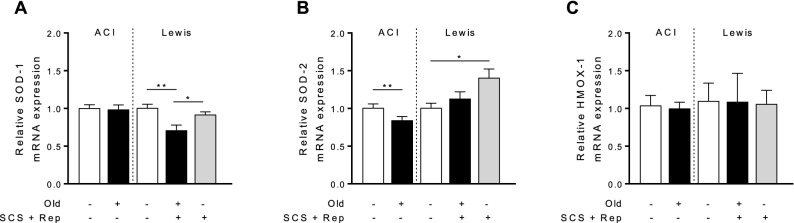
Antioxidant and cytoprotective gene expression. Tissue samples from old and young liver grafts following cold storage and reperfusion were assessed to determine the induction of antioxidant and cytoprotective genes. **a** Expression of superoxide dismutase-1 (SOD-1), the cytosolic form, is significantly lower in old liver grafts following cold storage and reperfusion. **b** SOD-2, the mitochondrial form, is significantly less in old untreated controls compared to young controls, indicating reduced expression at baseline. Following reperfusion, young liver grafts demonstrate significantly higher expression of SOD-2 while old liver grafts do not. **c** Heme oxygenase-1 (HMOX-1) expression was not significantly different between groups. Data shown as mean ± SEM, N = 4–6 per group, *p < 0.05, **p < 0.01.

Given the effect of age on sirtuin1 expression and the key role played by sirtuin1 in activating antioxidant defense mechanisms^19,20^, gene expression of antioxidant enzymes was compared in old vs. young liver grafts. Expression of superoxide dismutase-1 (SOD1), a cytosolic enzyme that acts on the superoxide anion, was significantly lower in old liver grafts in comparison to both young liver grafts and control liver tissue (Fig. 4a). Gene expression of SOD2, the mitochondrial form, was significantly upregulated in young, but not old, liver grafts in comparison to control liver tissue. SOD2 expression was lower in untreated old vs. young livers at baseline (Fig. 4b). Gene expression of heme-oxygenase-1, a cytoprotective enzyme involved in liver I–R, was not significantly different between groups (Fig. 4c).

### Mitochondrial quality control pathways are impacted by cold storage and reperfusion

**Figure 5:**
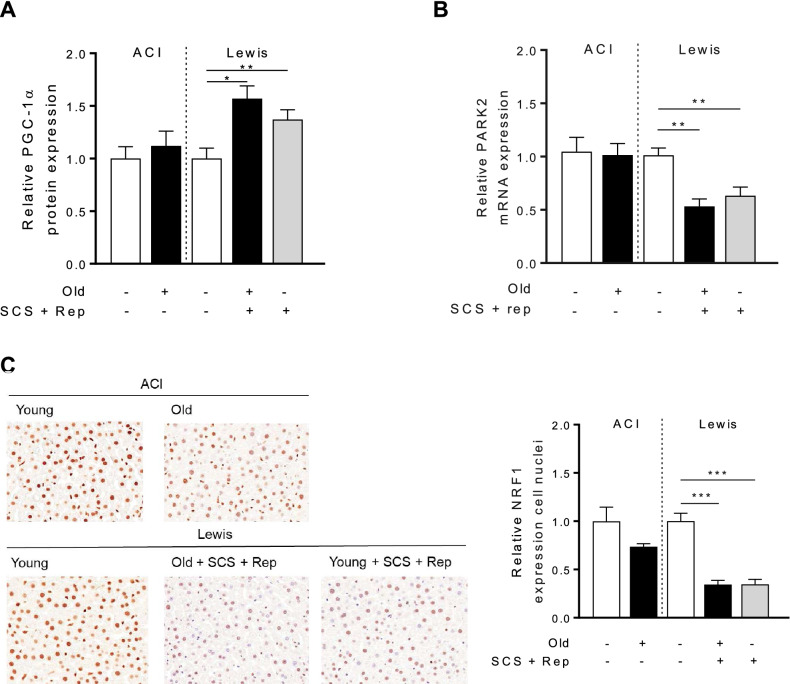
Mitochondrial quality control pathways. Tissue samples from old and young liver grafts following cold storage and reperfusion were assessed to determine levels of key regulators of mitochondrial biogenesis and mitophagy. **a** PGC-1α, an early activator of the mitochondrial biogenesis pathway, is greater in both old and young liver grafts following cold storage and reperfusion. **b** Gene expression of Parkin, an initiator of mitophagy, is lower in both old and young liver grafts following cold storage and reperfusion. **c** Nuclear expression of NRF-1, a downstream transcription factor in the mitochondrial biogenesis pathway, is significantly lower following cold storage and reperfusion, reflecting the lag between PGC1α activity and downstream NRF-1 expression. Data shown as mean ± SEM, N = 4–6 per group, *p < 0.05, **p < 0.01, ***p < 0.001.

Mitochondrial biogenesis and mitophagy are two key pathways involved in mitochondrial quality control^26^. In the biogenesis pathway, sirtuin1 functions as an activator of PGC1α, a key transcription factor that coordinates the nuclear and mitochondrial response^27^. To determine the impact of sirtuin1 on this pathway, levels of PGC1α were measured in graft tissue. In both old and young liver grafts, levels of PGC1α were increased relative to control liver tissue, indicating the activation of mitochondrial biogenesis pathways (Fig. 5a). Interestingly, expression of a downstream transcription factor, NRF-1, was significantly reduced for both old and young liver grafts following ischemia–reperfusion (Fig. 5b). To assess changes in mitophagy, gene expression of Parkin was compared for graft and control liver tissue. Parkin is responsible for the ubiquitylation of outer mitochondrial membrane proteins and initiation of mitophagy^28^. Interestingly, in both old and young livers grafts, Parkin expression was significantly decreased following cold storage and reperfusion (Fig. 5b).

### Transmission electron microscopy (TEM) demonstrates morphologic differences between old and young mitochondria

**Figure 6:**
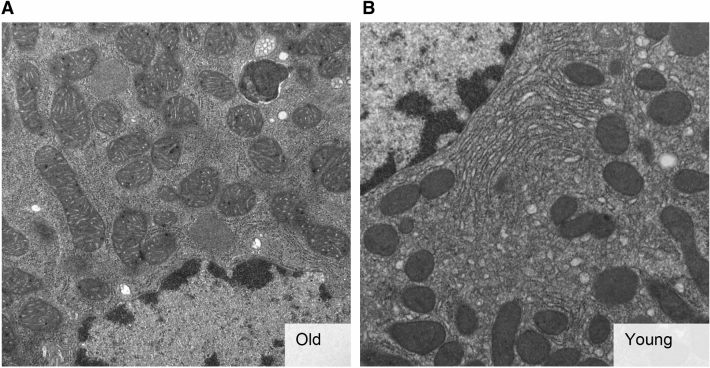
Transmission electron microscopy of hepatic mitochondria. Transmission electron microscopy of representative sections of **a** old liver grafts and **b** young liver grafts after 12 h of static cold storage and 2 h of reperfusion. The cristae of old mitochondria are swollen, but typical signs of mitochondrial damage including swelling of the mitochondria themselves, are not seen. Magnification: 11,500x.

TEM was performed to assess whether any morphologic differences exist between old and young mitochondria exposed to ischemia–reperfusion. TEM analysis demonstrated swelling of cristae in old mitochondria. More prominent signs of mitochondrial damage, including organelle swelling, were absent (Fig. 6).

### Old liver grafts sustain greater injury during cold storage, reflected by DAMP release

**Figure 7:**
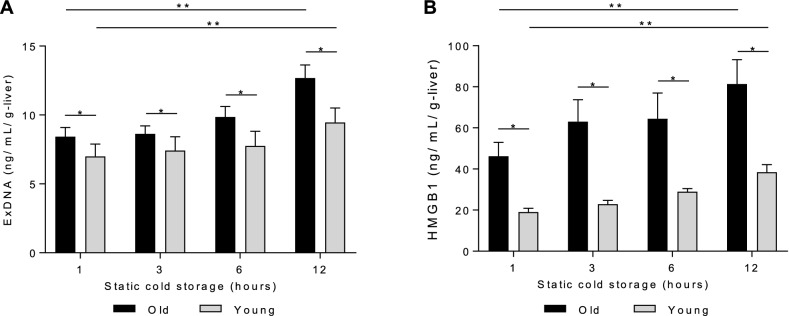
Release of DAMPs during static cold storage of old and young liver grafts. During 12 h of static cold storage in University of Wisconsin solution, levels of extracellular DNA (exDNA) and HMGB1 were measured in preservation solution. Levels of **a** extracellular DNA (exDNA) and **b** HMGB1 increased over time in both groups, but were significantly higher for old liver grafts, indicating more severe cold storage injury. Data shown as mean ± SEM, N = 7 per group, *p < 0.05.

To assess graft injury during cold storage, levels of exDNA and HMGB1, two DAMPs associated with ischemic liver injury^29,30^, were measured at 1 h, 3 h, 6 h, and 12 h (Fig. 7). Both ExDNA and HMGB1 levels were significantly higher for old liver grafts in comparison to young liver grafts at all time points. For both old and young liver grafts, exDNA and HMGB1 levels increased significantly from the 1 h time point to the 12 h time point, indicative of ongoing tissue injury.

### Old liver grafts demonstrate impaired metabolic function and increased injury during reperfusion

**Figure 8:**
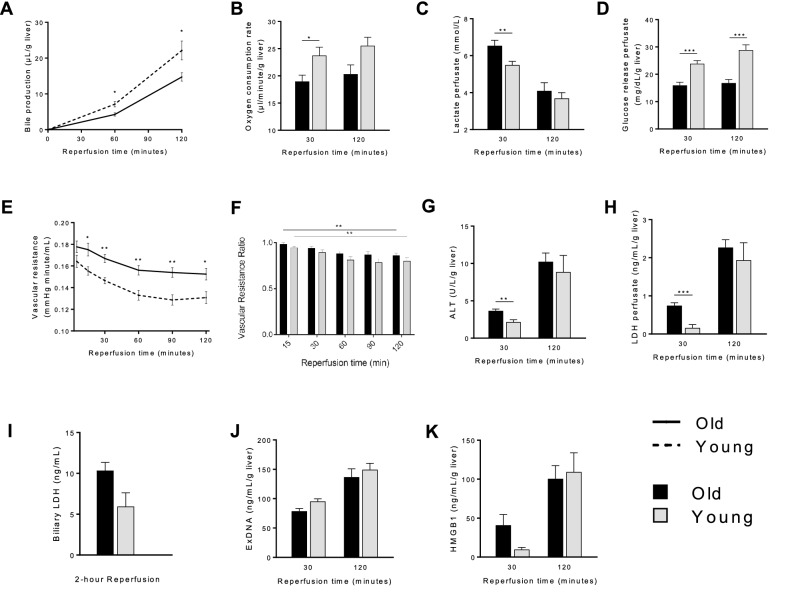
Graft function and injury during reperfusion of old and young liver grafts. After 12 h of static cold storage in University of Wisconsin solution, old and young liver grafts underwent ex vivo reperfusion for 2 h at 37 °C using Krebs–Henseleit buffer. **a** Bile production was significantly lower for old liver grafts, indicating decreased synthetic function. **b** The oxygen consumption rate of old liver grafts was significantly lower during the early reperfusion period, reflecting a lower graft metabolic rate. **c** Lactate levels in the perfusate were significantly higher for old liver grafts during the early reperfusion period, indicating impaired metabolic function. **d** Glucose release into the perfusate was significantly lower for old grafts, indicating impaired gluconeogenesis and glycogenolysis. **e** Vascular resistance during reperfusion was higher for old grafts, indicative of microvascular injury. **f** However, the vascular resistance ratio was similar between old and young liver grafts over the course of reperfusion. Markers of hepatocellular injury including **g** ALT and **h** LDH were significantly higher for old liver grafts in the early reperfusion period. **i** Biliary LDH levels, indicative of injury to biliary epithelium, were not significantly different between old and young grafts. Levels of **j** extracellular DNA (exDNA) and **k** HMGB1 released into perfusate were not significantly different between groups. Data shown as mean ± SEM, N = 7 per group, *p < 0.05, **p < 0.01, ***p < 0.001.

Impaired metabolic function of older grafts was demonstrated by several parameters, including lower bile production, reduced oxygen consumption, and higher perfusate lactate in comparison to young liver grafts (Fig. 8a–c). Old grafts also demonstrated impaired gluconeogenesis and glycogenolysis, evidenced by significantly lower glucose release into the perfusate (Fig. 8d). In addition, old liver grafts demonstrated increased injury during reperfusion, evidenced by higher vascular resistance (Fig. 8e), higher perfusate ALT (Fig. 8g), and higher perfusate LDH at 30 min (Fig. 8h). Other markers of injury were not significantly different between old and young liver grafts, including vascular resistance ratio (Fig. 8f), biliary LDH (Fig. 8i), perfusate exDNA (Fig. 8j), and perfusate HMGB1 (Fig. 8k). ExDNA levels were markedly increased (fivefold or more) during reperfusion compared to the end of SCS, while HMGB1 levels were similar between the reperfusion and SCS phases (Fig. 7 and Fig. 8j, k).

### Release of inflammatory molecules and induction of inflammatory genes does not differ between old and young liver grafts

**Figure 9:**
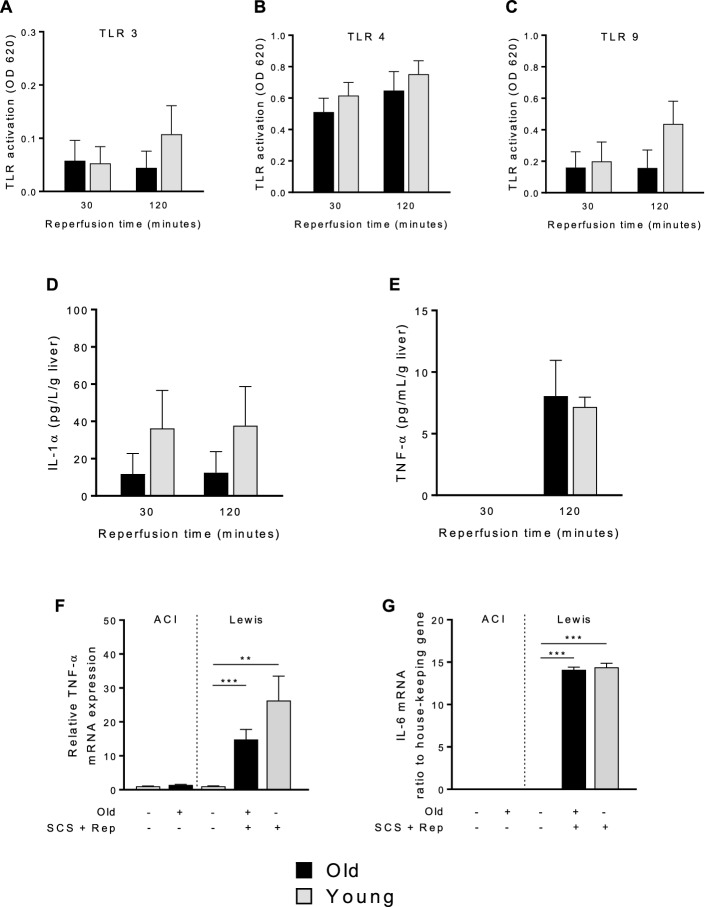
Inflammatory profile of old and young liver grafts. Perfusate samples were collected during reperfusion and Toll-like receptor (TLR) activation was measured by incubating perfusate with TLR reporter cell lines. Levels of inflammatory cytokines released into perfusate were measured by cytometric bead array. Tissue samples of liver grafts at the end of reperfusion and untreated control tissue were assessed for inflammatory gene expression. **a** TLR3, **b** TLR4, and **c** TLR9 activation by perfusate from old versus young liver grafts was not significantly different, indicating that liver grafts release comparable levels of inflammatory mediators during reperfusion. Similarly, levels of inflammatory cytokines **d** IL-1α and **e** TNF-α released into the perfusate were not significantly different between old versus young liver grafts. Inflammatory gene expression of **f** TNF-α and **g** IL-6 were not significantly different between old and young liver grafts, but significantly higher than untreated control livers. Data shown as mean ± SEM, N = 4–7 per group, *p < 0.05, **p < 0.01, ***p < 0.001.

To assess the proinflammatory milieu associated with reperfusion of old and young grafts, the release of TLR agonists and inflammatory cytokines was measured during reperfusion. There were no significant differences between old and young liver grafts in release of TLR-stimulating molecules (Fig. 9a–c). Similarly, there were no significant differences in the release of IL-1α or TNFα during reperfusion (Fig. 9d, e). Following reperfusion, gene expression of the inflammatory cytokines TNFα and IL-6 was significantly upregulated in both old and young liver grafts, but no significant differences existed based on age (Fig. 9f, g).

### Histologic injury and degree of apoptosis do not significantly differ between old and young liver grafts

**Figure 10:**
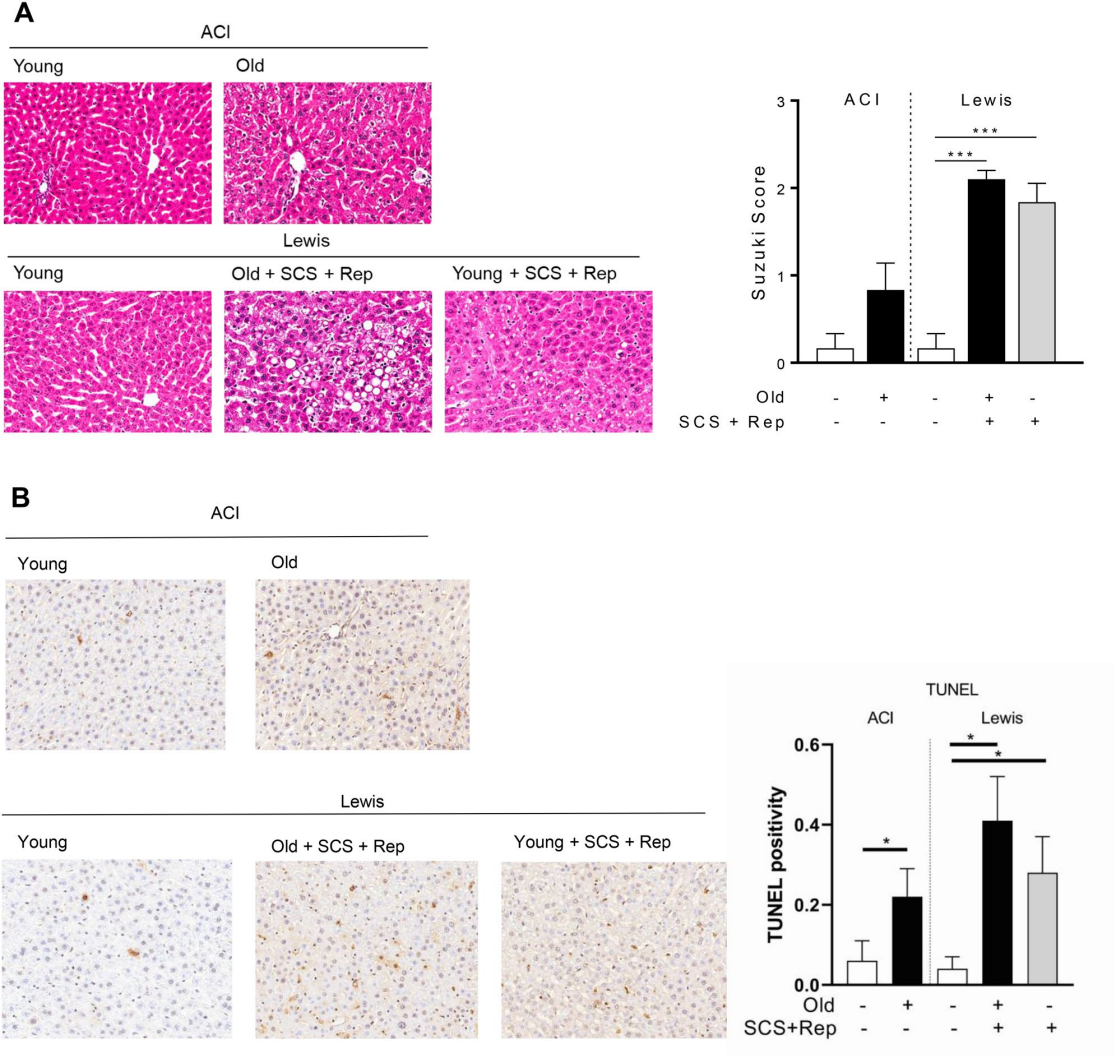
Histologic injury and apoptosis following graft reperfusion. Tissue samples from old and young liver grafts following cold storage and reperfusion were assessed to determine histologic injury by H&E and degree of apoptosis by TUNEL staining. **a** H&E staining demonstrates significantly increased hepatocyte vacuolization, congestion, and necrosis at the end of reperfusion in both old and young grafts, reflected in the Suzuki score. **b** TUNEL staining demonstrates significantly more apoptosis in liver grafts compared to control tissue, but no significant difference between old and young liver grafts. Data are shown as mean ± SEM, N = 4–6 per group, *p < 0.05, ***p < 0.001.

Following graft reperfusion, histologic analysis was performed to assess degree of graft injury. Both old and young liver grafts demonstrated significantly greater histologic injury compared to control liver tissue as assessed by Suzuki score (Fig. 10a), but there was no significant difference based on age. To assess the degree of apoptosis in the tissues, TUNEL staining was performed. Both old and young liver grafts demonstrated significantly greater apoptosis compared to control liver tissue, but again no significant differences were observed based on age (Fig. 10b).

## Discussion

The underlying cellular mechanisms contributing to increased susceptibility of old liver grafts to I–R injury have not been fully elucidated. Recent mechanistic studies indicate that diminished sirtuin-1 activity in aged hepatocytes contributes to an impaired mitochondrial response to I–R injury in vitro^16^, but the relevance of this pathway in liver transplantation remains unknown. In this study, we demonstrate that old liver grafts exhibit decreased expression and activity of sirtuin-1 (Fig. 2), which correlates with impaired mitochondrial function (Fig. 3), and ultimately impaired graft metabolic function (Fig. 8). The findings in our study are consistent with a recent study by Nakamura and colleagues who demonstrated that increased sirtuin-1 protein expression in post-reperfusion liver biopsies was associated with reduced early allograft dysfunction and enhanced graft survival in human liver transplants^31^. Due to limited sample size, however, Nakamura was unable to demonstrate an association between donor age and sirtuin-1 expression.

From a mechanistic perspective, the effects of sirtuin1 on mitochondrial function are mediated in part by activation of antioxidant defense mechanisms^19,20^ and mitochondrial biogenesis pathways^27^. In this study, we demonstrate a correlation between diminished sirtuin1 in old liver grafts and impaired antioxidant gene expression (Fig. 4), but did not observe any impairment in mitochondrial biogenesis activity (Fig. 5). The latter observation may be due to the relatively early time point at which we made our assessment (2 h post reperfusion). Importantly, the diminished function of old grafts does not appear to be driven by greater inflammation (Fig. 9) or increased histologic injury (Fig. 10). Our study also confirms the increased susceptibility of old liver grafts to cold storage preservation injury. Release of extracellular DNA and HMGB1, two DAMPs indicative of cell death, were significantly elevated in the cold storage preservation solution of old liver grafts (Fig. 7). This finding is consistent with clinical reports demonstrating an association between shorter cold ischemic time and improved outcomes with older donor grafts^2^. This concept also supports the potential use of normothermic machine perfusion for the preservation of old grafts to reduce exposure to cold ischemia.

There are some important limitations of this study that should be recognized. Due to the large size of the old liver grafts, it was not technically feasible to perform orthotopic liver transplants into younger recipients. As such, graft reperfusion was performed by ex vivo reperfusion with an acellular perfusate (Krebs–Henseleit buffer). We selected this model because it is a standardized approach for ex vivo liver reperfusion experiments in the literature^32–37^, but acknowledge that liver transplantation is the optimal reperfusion model. Second, we assessed mitochondrial function indirectly by measuring ATP and ADP levels in the tissue. While this is a standard approach in ischemia–reperfusion models^14,17^, alternative techniques to directly measure mitochondrial function such as high resolution respirometry may provide more precision. Finally, due to the limited availability of old rats, we included 2 strains (Lewis and ACI), with the ACI strain serving as the untreated control. The high level of concordance observed between these 2 strains in the young cohort is reassuring that this comparison is valid.

In conclusion, this study supports the emerging importance of sirtuin-1 in liver transplantation and highlights age-related differences that may contribute to the discrepancy in outcomes between young and old liver grafts. The delivery of pharmacologic activators of sirtuin-1 during machine perfusion may represent a therapeutic strategy to improve the function of old liver grafts.

## Data availability

The datasets generated during the current study are available from the corresponding author on reasonable request.

## Abbreviations

ACI: August Copenhagen Irish
ECD: Extended criteria donor;
exDNA: Extracellular DNA
HEK: Human embryonic kidney
HMGB1: High-mobility group box 1
I–R: Ischemia-reperfusion
LDH: Lactate dehydrogenase
LT: Liver transplantation
PGC1α: Peroxisome proliferator-activated receptor gamma coactivator 1-alpha
SCS: Static cold storage
SEAP: Secreted embryonic alkaline phosphatase
SOD: Superoxide dismutase
TLR: Toll-like receptor
TNF: Tumor necrosis factor
